# Coexistence of Sjögren syndrome in patients with synovitis, acne, pustulosis, hyperostosis, and osteitis syndrome: A retrospective observational study

**DOI:** 10.1097/MD.0000000000023940

**Published:** 2021-03-26

**Authors:** Lun Wang, Yanying Yu, Shuo Zhang, Wen Zhang, Chen Li

**Affiliations:** a Institute of Clinical Medicine; b Department of Rheumatology and Clinical Immunology, Peking Union Medical College Hospital; c Department of Traditional Chinese Medicine, Peking Union Medical College Hospital, Chinese Academy of Medical Sciences & Peking Union Medical College, Beijing, China.

**Keywords:** acne, and osteitis syndrome, clinical characteristic, hyperostosis, prevalence, pustulosis, Sjögren syndrome, synovitis

## Abstract

To identify the prevalence and clinical characteristics of Sjögren syndrome (SS) in a Chinese single-center cohort of synovitis, acne, pustulosis, hyperostosis, and osteitis (SAPHO) syndrome.

Patients diagnosed with SS were screened out from a cohort of 164 cases of SAHPO syndrome. Information regarding the patients’ gender, age at onset, clinical features, laboratory tests, bone scintigraphy, and treatment was reviewed.

Five patients were screened out. The prevalence of SS in SAPHO patients was 3.05% The mean onset age of SS was 48.0 ± 12.0 years old and no apparent time order in the occurrence of SAPHO and SS was observed. Compared with the general SAPHO cohort, the 5 SS patients exhibited no significant difference in the SAPHO related clinical features or inflammatory markers, except for a higher prevalence of peripheral joints and bones involvement in bone scintigraphy. Objective evidence of dryness and positive salivary gland biopsy were found in all the patients. However, the positive rates of SSA and SSB antibody were only 20%. Anti-inflammatory treatment for SS was recorded in 3 patients (ESSDAI score: 3 in 2 patients; 12 in 1 patient) with extra-glandular manifestations, severe complications or poor response to the basic treatment.

The prevalence of SS is higher in the SAPHO cohort than in the general Chinese population. Objective tests or biopsy might be more indicative than the antibody detection for SS diagnosis. Anti-inflammatory treatment should be prescribed in consideration of both the severity of SS and the demand for disease activity control of SAPHO.

## 1 Introduction

Synovitis, acne, pustulosis, hyperostosis, and osteitis (SAPHO) syndrome is a rare disease characterized by its osteoarticular and dermatological disorders.^[1]^ To date, the etiology of SAPHO remains unclear, while numerous evidences have supported that immune dysfunction plays an important role in the pathogenesis. SAPHO is currently considered as an autoimmune disease. Immunosuppression drugs have been wildly used and achieved significant efficacy.^[1,2]^

Other autoimmune diseases have been identified in patients with SAPHO syndrome in the form of case reports, such as inflammatory bowel disease and rheumatoid arthritis.^[3–5]^ Sjögren's syndrome (SS) is a common autoimmune disease featured by lymphocytic infiltration of the secretory glands and resulting in glandular dysfunction. However, it has been reported that the diagnosis of SS is often delayed.^[6]^ So far, no case has been identified in the patients with SAPHO syndrome. Sjögren's syndrome (SS) may have been overlooked in patients with SAPHO syndrome. The clinical features of the patients with the coexistence of SAPHO and SS remain unclear.

Our study aimed to identify the prevalence of SS in patients with SAPHO syndrome and demonstrate their clinical characteristics.

## 2 Patients and methods

We performed a retrospective observational study of patients who had the concurrence of SS among a Chinese single-center cohort of 164 cases of SAPHO syndrome published previously.^[7]^ All the patients fulfilled the diagnostic criteria for SAPHO syndrome proposed by Kahn and Khan.^[8]^ We thoroughly reviewed the medical history of all the patients and included patients who had been diagnosed with SS before recruited to the cohort and during the follow-up. The diagnosis of SS was established according to the 2002 American-European Consensus Group criteria,^[9]^ or 2012 ACR criteria^[10]^ according to the record. We reevaluated and confirmed the diagnosis based on the 2016 American-European Consensus Sjögren's Classification Criteria (ACR/EULAR).^[11]^

The study was in compliance with the Declarations of Helsinki and was approved by the Ethics Committee of Peking Union Medical College Hospital, Peking Union Medical College and Chinese Academy of Medical Sciences (Identifier: ZS-944). Informed written consent was obtained from each patient.

Information regarding the patients’ gender, age at onset, clinical features, laboratory tests, bone scintigraphy, and treatment for SAPHO and SS was reviewed. The results of examinations related to the diagnosis of SS including whole sialometry, Schirmer test, labial salivary gland biopsy and antibody tests of antinuclear antibody (ANA), anti-SSA, and anti-SSB were recorded. The EULAR Sjögren syndrome disease activity index score was also evaluated.^[12,13]^

Continuous data are presented as mean, and categorical data are displayed as numbers (n) or proportions (%). Differences between SAPHO cohort and patients with SAPHO and SS were analyzed for significance using a 2 independent samples t-test or a non-parametric test (continuous data) and Fisher exact test (categorical data). The statistical significance was defined as *P* < .05. Statistical analysis was conducted using SPSS 19.0 (IBM, Chicago).

## 3 Results

### 3.1 Baseline characteristics

Five patients (3.05%) who met the diagnostic criteria of both SAPHO and SS were screened out from the 164-patient cohort. The demographic and clinical characteristics of the whole cohort and the patients with coexisting SS were summarized in Table 1. All five patients were female. The rheumatoid factor and human leukocyte antigen B27 were negative in all the five patients and no other concomitant rheumatic diseases, including but not limited to rheumatoid arthritis or systemic lupus erythematosus, were recorded. Four out of five patients were positive for the ANA test, which was significantly higher than the rate of SAPHO cohort (*P* < .01). Besides, according to the result of whole-body bone scintigraphy, compared with the general SAPHO cohort, patients with SAPHO and SS seem to have a higher prevalence of peripheral joints and bones involvement which is mainly contributed from an increasing ratio of foot lesions. No other significant difference in the SAPHO related clinical features or the inflammatory markers (erythrocyte sedimentation rate and high-sensitivity C-reactive protein) was found between the five patients and the whole cohort.

**Table 1:** Demographic and clinical characteristics of the SAPHO cohort and patients with SAPHO and Sjögren's syndrome.

	SAPHO cohort (n = 164)	Patients with SAPHO and SS (n = 5)	*P* value
Age at onset of SAPHO symptoms, yr	36.8 (10.8)	44.0 (17.9)	.16
Gender, female/male	111/53	5/0	.30
Clinical characteristics			
Osterarticular lesions	164 (100)	5 (100)	NA
ACW	164 (100)	5 (100)	NA
PJB	136 (67.7)	4 (80.0)	>.99
Whole-body bone scintigraphy	(n = 157)	(n = 5)	
ACW	156 (99.4)	5 (100)	>.99
Sternocostoclavicular region	144 (91.7)	5 (100)	>.99
Anterior Rib(s)	73 (46.5)	3 (60.0)	.89
Posterior Rib(s)	9 (5.7)	0 (0)	>.99
Axial skeleton	93 (59.2)	5 (100)	.17
Spine	71 (45.2)	5 (100)	.05
Sacroiliac joint(s)	46 (29.3)	1 (20)	>.99
PJB	54 (34.4)	5 (100)	.01
Shoulder	11 (7.0)	1 (20)	.32
Elbow	3 (1.9)	0 (0)	>.99
Wrist	3 (1.9)	0 (0)	>.99
Hand	5 (3.2)	0 (0)	>.99
Hip	5 (3.2)	0 (0)	>.99
Knee	20 (12.7)	1 (20.0)	.51
Ankle	11 (7.0)	1 (20.0)	.32
Foot	22 (14.0)	3 (60.0)	.03
Long bones	9 (5.7)	1 (20.0)	.28
Pelvis	4 (2.5)	0 (0)	>.99
Skull	16 (10.2)	2 (40.0)	.10
Skin manifestations	155 (94.5)	4 (80.0)	.27
PPP	143 (84.6)	4 (80.0)	.51
SA	25 (15.2)	0 (0)	>.99
PPP and PV	23 (14.0)	1 (20.0)	.54
Laboratory tests			
ANA	12 (7.3)	4 (80.0)	<.01
RF	2 (1.2)	0 (0)	>.99
HLA-B27	4 (2.4)	0 (0)	>.99
hsCRP	114/161 (70.8)	5 (100)	.36
ESR	93/163 (57.1)	4 (80.0)	.57

Data are presented as mean (SD) or n (%). ACW = anterior chest wall, ANA = anti-nuclear antibody, ESR = erythrocyte sedimentation rate, hsCRP = high-sensitivity C-reactive protein, HLA-B27 = human leukocyte antigen B27, NA = not applicable, PJB = peripheral joints and bones, PPP = palmoplantar pustulosis, PV = psoriasis vulgaris, RF = rheumatoid factor, SA = severe acne.

Table 2 shows the detailed clinical characteristics of the patients with SAPHO syndrome and coexisting SS. Among the five patients, the mean ages at the onset of SAPHO syndrome and SS were 44.0 ± 17.9 and 48.0 ± 12.0 years, respectively. There was no apparent time order in the occurrence of SAPHO and SS. Two patients had SAPHO manifestations before SS symptoms, whereas 2 and one patients had SAPHO manifestations after and at the same time as SS symptoms, respectively.

**Table 2:** Clinical characteristics of the patients with SAPHO syndrome and coexisting Sjögren syndrome.

	Patient 1	Patient 2	Patient 3	Patient 4	Patient 5
Gender	Female	Female	Female	Female	Female
Age at onset of SAPHO (yr)	13	45	52	52	58
Age at onset of SS (yr)	55	45	51	29	60
Interval (yr)	42	0	−1	−23	2
SAPHO symptoms					
Osteoarticular pain	ACW+AS+PJB	ACW+AS+PJB	ACW+AS+PJB	ACW	ACW+AS+PJB
Peripheral joint pain	Bilateral shoulders; Right hip	Bilateral shoulders; Right ankle	Bilateral shoulders	None	Bilateral shoulders
Skin lesions	PPP+PV	PPP	PPP	None	PPP
SS symptoms					
Dry mouth	+	+	+	+	+
Dry eye	+	+	+	+	+
Salivary gland enlargement	−	−	−	Bilateral	−
Fever	−	−	+	+	+
Leukopenia	−	−	−	+	−
Thyroid disease	+	−	−	−	+
Dental caries	−	+	−	+	−
Canker sores	−	+	−	+	+
Conjunctivitis	−	−	−	+	−
ESSDAI scores	0	0	3	12 (IgG>20g/L)	3
SS diagnostic tests					
Whole sialometry	+	−	+	+	+
Parotid gland contrast sialography	+	−	+	+	N
Labial salivary gland biopsy	+	+	+	+	+
Schirmer test	+	+	+	+	+
Tear break-up time	N	+	N	N	N
ANA	1:640	1:80	1:160	1:320	−
SSA	−	−	−	+	−
SSB	−	−	−	+	−
Treatment					
SAPHO treatment	NSAIDs; Bisphosphonates; TwHF	NSAIDs; Bisphosphonates; MTX	NSAIDs; TNFi	NSAIDs; Leflunomide	NSAIDs
SS treatment	Basic self-care measures; Artificial tears	Basic self-care measures; Artificial tears	Basic self-care measures; Artificial tears; Glucocorticoids	Basic self-care measures; Artificial tears; Glucocorticoids	Basic self-care measures; Artificial tears; Glucocorticoids; HCQ

ACW = anterior chest wall, ANA = anti-nuclear antibody, AS = axial skeleton, ESSDAI = EULAR Sjögren's syndrome disease activity index, HCQ = hydroxychloroquine, MTX = ethotrexate, N = not tested, NSAIDs = non-steroidal anti-inflammatory drugs, PJB = peripheral joints and bones, PPP = palmoplantar pustulosis, PV = psoriasis vulgaris, SSA = anti-Ro/SSA antibody, SSB = anti-La/SSB antibody, TNFi = tumor necrosis factor-α inhibitors, TwHF = *Tripterygium wilfordii* hook f.

### 3.2 Clinical features and laboratory tests

In terms of SAPHO-related syndromes, all patients suffered from anterior chest wall pain. The osteoarticular pain of the axial skeleton together with peripheral joints and bones was found in 4 patients but none of the five patients complained about morning stiffness of the peripheral joints. Skin lesions were found in 4 patients, including palmoplantar pustulosis in all of the patients and psoriasis vulgaris in 1 patient. As for SS-related syndromes, all patients presented typical clinical symptoms of xerophthalmia and xerostomia. Three patients (60%) presented a low-grade fever. One patient had bilateral enlargement of the salivary gland. Thyroid disease was presented in 2 patients and leukopenia in 1 patient. In terms of complications of mucosal dryness, 2 patients presented dental caries and 3 presented recurrent canker sores. No other extra-glandular involvement was observed, including arthritis, vasculitis, neurological, renal or pulmonary involvement.

Objective evidence of both salivary gland and ocular involvement was found in all the five patients. Four patients have undertaken parotid sialography with positive results in 3 of them (3/4). Four patients were positive for the whole sialometry. Meanwhile, focal lymphocytic sialadenitis was found in the salivary glands of all the 4 patients undertaking salivary gland biopsy. Besides, all patients presented decreased lacrimal gland secretion by the Schirmer test.

For the laboratory findings, 4 out of 5 patients were positive for the ANA test with the titer ranging from 1:80 to 1:640. Nevertheless, only one of these patients (1/5) was positive for both anti-SSA antibody and anti-SSB antibody and the other patients (4/5) were negative for either anti-SSA antibody or anti-SSB antibody.

### 3.3 Treatment

All the patients have received treatment for SAPHO and SS. A multidisciplinary approach was made among ophthalmology, stomatology, and rheumatology specialists for the treatment strategies of these patients. All patients received comprehensive patient education of self-care strategies and suggestions on effective use of artificial tears. For 3 patients who had high severity, including extra-glandular manifestations, severe complications of mucosal dryness or poor response to basic treatment, systematic anti-inflammatory treatment of glucocorticoids alone (Patient 3: prednisone, initial dosage of 55 mg/d for a month, and was gradually reduced until final cessation after 9 months (before the onset of SAPHO); Patient 4: prednisone, initial dosage of 30 mg/d for a month, and was gradually reduced until final cessation after 6 months (before the onset of SAPHO)) or glucocorticoids plus hydroxychloroquine (Patient 5: prednisone, 10 mg/day for a month; hydroxychloroquine, 20 mg/d) was prescribed. All these 3 patients had a fever during the onset of SS.

## 4 Discussion

SAPHO syndrome is characterized by a collection of dermatological and osteoarthritic disorders with pathogenesis involving infectious, immunologic and genetics factors that leading to the activation of the innate and cell-mediated immune response.^[1,13]^ However definite causes of SAPHO syndrome have not been fully understood.

A previous study has demonstrated a high prevalence of autoimmune disease in a Dutch cohort of 70 cases of sternocostoclavicular hyperostosis which is considered as a part of SAPHO syndrome, but SS was only reported in 1 patient.^[14]^ The coexistence of SAPHO syndrome and other autoimmune diseases has also been reported in the form of case reports. Nissen et al. reported a 68-years old female patient who initially diagnosed seropositive rheumatoid arthritis concurrently satisfied the diagnosis criteria of SAPHO syndrome.^[5]^ Xu et al. also described a clinical case of coexistence of SAPHO syndrome and rheumatoid arthritis with an onset interval of 10 years.^[4]^ These cases indicated that it is possible for the co-occurrence of autoimmune diseases in patients with SAPHO syndrome. Here, we first report 5 patients with concurrence of SAPHO syndrome and SS.

SS has an estimated prevalence of 0.45% to 0.77% in the Chinese reference population.^[15,16]^ While our study showed that the prevalence of SS in our cohort of SAPHO patients was 3.05%, which was much higher than the prevalence in the Chinese general population.

Several researches revealed the potential pathogenesis of SAPHO syndrome and SS. Common autoimmune process has been discovered between these 2 different diseases. Studies showed that the Th1 cell cytokines including interleukin (IL)-6 and TNF-α were evaluated both in the salivary of patients with primary SS and in the serum of patients with SAPHO syndrome.^[17,18]^ For the IL-23-IL23R-Th17-IL17 axis, Th17 cells were reported being increased in the peripheral blood in patients with SAPHO syndrome and in the salivary gland biopsy in patients with primary SS. Meanwhile, the secretion of IL-17 was also increased in both diseases.^[19,20]^ Furthermore, IL-8, a cytokine that induces the secretion of interferon γ, was also proved to be evaluated in the patients of both diseases. These findings indicated that there are common immunological processes that participate in the occurrence of SAPHO syndrome and SS, which might be a possible explanation for the coexistence and higher prevalence of SS in the patients with SAPHO syndrome. Further analysis of T/B lymphocyte subsets, cytokines, chemoattractants, and pro-inflammatory small molecules from the peripheral blood of patients with both SS and SAPHO syndrome and those with either disease might help to uncover the common immunological processes, and identify potential indicators for disease monitoring and also targets for treatment.

This finding supported the importance of genetic predisposition to autoimmune diseases in patients with SAPHO syndrome. In the Dutch study of sternocostoclavicular hyperostosis, Valkema et al.^[14]^ demonstrated that the prevalence of autoimmune disease was increased in both the patients and their first-degree relatives. On the other hand, another possible reason for the higher prevalence may lie in the specific age and gender of the patients. In SAPHO cohort, the mean age was 40.7 years and the number of women was as twice as of men, both of which may increase the prevalence of SS.

SAPHO syndrome is to some extent considered as a subtype of spondyloarthritis (SpA).^[21]^ The coexistence of SS and SpA were reported in previous studies,^[22,23]^ and in a study involving 70 patients with ankylosing spondylitis, researchers found a high prevalence of concomitant SS (10%).^[24]^ It was showed that SpA patients with coexisting SS share features, including old age, female, high frequency of positive ANA, and sicca syndrome, and they do not seem to exhibit a more severe spondyloarthropathy.^[25]^ Our results are consistent with these findings; besides, we further found that the clinical characteristics of patients with SS and SAPHO syndrome in not exactly identical to that of the general SS or SAPHO patients.

The osteoarticular lesions are the fundamental inflammatory change in SAPHO syndrome. Among the extra-glandular systemic manifestations, joint involvement is also observed in 20–60% of primary SS (pSS) patients, and among them one third of patient present synovitis but there is usually no joint erosion. Besides, cyclic citrullinated peptide-antibodies were also reported to in 5–10% pSS patients.^[26]^ The articular manifestations in patients with pSS could share similarity with rheumatoid arthritis but differ by the absence of structural damage.^[27]^ We have observed that patients with SAPHO and SS seem to exhibit higher ratio of peripheral joint and bone involvement in bone scintigraphy than the general SAPHO patients. However, the typical symmetrical involvement and morning stiffness of multiple peripheral synovial joints was not observed in the present study. Therefore, the level of cyclic citrullinated peptide antibody -antibodies and synovitis evaluated radiographically or by ultrasonography in both the general SAPHO patients and those with coexisting SS need to be clarified in further studies to identify the pathological changes of peripheral joints.

Patients with coexistence of SAPHO syndrome and SS have unique characteristics comparing to the general population with SS. All the patients had symptoms of dryness and positive biopsy findings, which differs from the conclusion drawn from previous reports that Asians present low rate of dryness symptoms. Moreover, while anti-SSA and anti-SSB are auto-antibodies wildly used in the diagnosis of SS in various classification criteria^[11]^ with an average estimated prevalence of 63% and 40%, respectively. Our study exhibited only a 20% prevalence of anti-SSA and anti-SSB antibody in patients with coexistence of SAPHO syndrome and SS. This indicated that the pathophysiology of SS in patients with SAPHO syndrome is not thoroughly identical with that of primary SS. Besides, in terms of their low positive rate, the diagnostic value of anti-SSA and anti-SSB antibody should be reconsidered in patients with SAPHO syndrome. Objective tests of dryness and salivary gland biopsy might play a more important role and the results of multiple tests would always be required to confirm the diagnosis of SS in SAPHO patients with symptoms of dryness.

Similar to the treatment of the general SS population, self-management and artificial tears are always indispensable as basic measures. For patients with extra-glandular manifestations or multiple complications, systemic anti-inflammatory treatment should be considered. As it is found in our study that the patients with a fever during the onset of SS tend to have higher disease activity and poor response to the basic measures, systemic anti-inflammatory treatment may be frequently needed. In a previous prospective study, higher levels of beta2-microglobulin and free light chains of immunoglobulins in pSS patients are also associated with increased systemic disease activity, which could facilitate the evaluation of disease condition and further decision of treatment scheme.^[28]^ In the present study, 3 patients have received systemic anti-inflammatory treatment for SS. Two of the patients have finished the treatment before the onset of SAPHO syndrome and only 1 patient (Patient 5) started the treatment after the diagnosis of SAPHO syndrome. It is noteworthy that Patient 5 only received non-steroidal anti-inflammatory drugs for SAPHO management and such low-intensity treatment is not commonly observed in the follow-up of our cohort. It is possible that the corticosteroids and hydroxychloroquine prescribed for SS might also contribute to the disease activity control of SAPHO syndrome simultaneously. Therefore, it is important to take both SS and SAPHO syndrome into consideration and make a comprehensive treatment decision in the clinical practice. Joint efforts from ophthalmology, stomatology, and rheumatology specialists are needed to improve the management of these patients.

For the treatment of SS with high disease activity in SAPHO patients, in addition to the traditional systemic anti-inflammatory drugs, the novel targeted synthetic disease-modifying anti-rheumatic drugs, Janus Kinase (JAK) inhibitors, might serve as a promising solution to the simultaneous disease activity control of both diseases.^[29]^ Tofacitinib, a small-molecule nonspecific JAK1 and JAK3 inhibitor, was used to treat a refractory SAPHO patient and demonstrated amelioration in terms of clinical symptoms, inflammatory parameters and MRI.^[30]^ A pilot study was also conducted showing the effectiveness of tofacitinib in 12 patients with SAPHO syndrome, evidenced by the improvement of bone pain, rash, inflammatory markers, quality of life, and MRI evaluation.^[31]^ For the treatment of pSS, it is now proposed that JAK inhibition might also be a novel therapeutic approach for primary SS by suppressing the activation of salivary gland epithelial cells and controlling the oxidative stress.^[32,33]^ A clinical trial of filgotinib, a selective JAK1 inhibitor, is currently underway in patients with pSS and tofacitinib remains to be extensively tested in pSS.^[34]^ Besides, according to EULAR recommendations, the use of rituximab in pSS may be considered in patients with severe, refractory systemic disease, and that the best indication is probably for symptoms linked to cryoglobulinemic-associated vasculitis. Rituximab is also essential for the treatment of the worst complication of pSS, B-cell lymphoma.^[35,36]^ Therefore, for pSS patients with lymph node enlargement which might indicate chronic B cell activation, B cell depletion with rituximab should be considered. The use of rituximab in SAPHO syndrome has not been reported. Although the lack of autoantibodies does not support the B cell deletion treatment in SpA, some small prospective open-label studies have shown potential benefit.^[37]^ However, the general efficacy remains equivocal.^[38]^ The coexistence of SAPHO and SS can provide opportunities for future observational studies to explore the efficacy of JAK inhibitors in SS and rituximab in SAPHO syndrome.

The major limitation of the present study is that it is only a retrospective study of 5 cases. Potential deviation due to the small sample size has hindered further interpretation of the current results. To draw more solid conclusions on the actual prevalence and clinical characteristic of SS in SAPHO patients, future prospective study with larger sample size is required. Besides, long-term follow-up of the diagnosed patients should be performed to clarify the efficacy of treatments and prognosis. Multi-center collaborations might be helpful in reaching meaningful sample size and conclusive results. In addition to the coexistence of SS, future studies can summarize the prevalence of various other autoimmune diseases in patients with SAPHO syndrome. It is also important to conduct survey on their relatives, especially parents, siblings, and children, to clarify genetic predisposition and identify potential susceptible pedigree and genetic factors.

## 5 Conclusion

In conclusion, the prevalence of SS is higher in our single-center cohort of SAPHO syndrome than in the general Chinese population. Compared with SSA or SSB antibody testing, objective ophthalmic and dental tests, as well as glandular biopsy might be of greater diagnostic value for SS with comorbid SAPHO syndrome. Systemic anti-inflammatory treatment should be considered based on the evaluation of both the severity of SS and the demand for disease activity control of SAPHO.

## Acknowledgments

We appreciate Yihan Cao's help in statistical analysis.

## Author contributions

**Conceptualization:** Lun Wang, Wen Zhang, Chen Li.

**Data curation:** Yanying Yu.

**Writing – original draft:** Lun Wang, Yanying Yu.

**Writing – review & editing:** Shuo Zhang, Wen Zhang, Chen Li.
